# Evaluation of tube extender use in glaucoma management: insights from a cohort study

**DOI:** 10.1007/s00417-026-07180-w

**Published:** 2026-03-20

**Authors:** Alejandro Marin, Maria A. Quintero, Tyler M. Kaplan, Marie M. Macaron, Sonya Agapov, Elena Bitrian

**Affiliations:** 1 Bascom Palmer Eye Institute, Miami, FL USA; 2 University of Miami Miller School of Medicine, Miami, FL USA; 3 Pontificia Universidad Javeriana, Bogota, D.C Colombia

**Keywords:** Tube extender, Tube revision, Glaucoma drainage device, Glaucoma surgery

## Abstract

**Purpose:**

To describe the clinical indications for tube revision with a Tube Extender and to characterize the eyes in which they were used.

**Methods:**

Single-center retrospective cohort of eyes undergoing Tube Extender implantation (2015–2023). Clinical data were collected from medical records, and Fisher’s exact test assessed associations between glaucoma type and revision indication.

**Results:**

Twenty-two eyes from 22 patients were included. The mean age at tube revision was 53 years, and 68% of patients were male. The most common glaucoma types were primary open-angle glaucoma (36%), uveitic glaucoma (23%), and neovascular glaucoma (18%). Tube Extenders were most frequently used with Ahmed devices (59%), followed by Baerveldt devices (28%). The mean interval from the original aqueous shunt surgery to Tube Extender implantation was 764 days, with a median of 497 days (IQR 100–582). Eighteen tube revisions were performed for a single indication, while four were performed for two or more indications. The main reasons for implantation were tube retraction (41%), corneal touch (27%), tube exposure (23%), and tube obstruction (13%). The tube was most commonly repositioned back into the anterior chamber (64%), followed by repositioning from the anterior chamber to the sulcus (27%) and repositioning within the sulcus (9%). Out of the 22 tube revisions, two were combined with cataract surgery and one with corneal transplantation. There was no statistically significant association between glaucoma type and indication for tube revision.

**Conclusions:**

Tube Extenders were primarily used to address complications such as tube retraction, corneal issues associated with glaucoma drainage devices, tube exposure, and obstruction. They offer a practical surgical solution to salvage existing aqueous shunt implants without the need for full device replacement, but additional studies are needed to assess long-term outcomes.

## Key messages

*****What is known?***:**

- Tube position–related complications such as retraction, corneal touch, exposure, and obstruction are common causes of failure after glaucoma drainage device surgery.
- Several surgical techniques exist to manage these complications, including tube trimming, tube-in-tube techniques, angiocatheter extensions, and commercially available Tube Extenders.

*****What is new?***:**

- This study provides the first systematic, cohort-based characterization of the clinical indications leading to Tube Extender use in glaucoma drainage device revision surgery.
- No significant association was found between glaucoma subtype and indication for Tube Extender revision, suggesting that Tube Extender use is primarily driven by mechanical and anatomic factors rather than disease etiology.

## Introduction

Glaucoma drainage devices (GDDs) are an established surgical option for patients with refractory glaucoma, particularly when trabeculectomy has failed or is unlikely to succeed. Concern about complications related to blebs has also led to the increased use of tube shunts as an alternative to trabeculectomy [1, 2]. These devices consist of an explant plate that, once encapsulated, forms a reservoir for aqueous humor drainage through a connected silicone tube, offering benefits comparable to trabeculectomy in terms of intraocular pressure (IOP) control and duration of effect^3^. Despite their effectiveness, complications such as hypotony, excessive capsule fibrosis, strabismus, infection, plate-edge erosion, and tube position–related problems may result in surgical failure [3–6].

To address tube position–related challenges, the main management options include placement of a 22-gauge angiocatheter, use of a tube-in-tube technique in which a trimmed segment of a glaucoma drainage device is inserted into the lumen of the existing tube, and the use of a commercially available Tube Extender (New World Medical, Rancho Cucamonga, CA) [7–11]. The Tube Extender is compatible with glaucoma drainage devices from different manufacturers, providing a convenient alternative to surgeon-fashioned extensions when tube lengthening is required [12, 13]. However, published information on Tube Extender use remains limited, and most publications emphasize the surgical pearls [8, 9, 12–14] or outcomes [11, 13] rather than systematically evaluating indications or patient characteristics. To address this gap, we conducted a retrospective cohort study to describe the demographic and clinical features of patients undergoing Tube Extender implantation, evaluate the indications leading to their use, and characterize final tube repositioning sites.

## Methods

This was a retrospective cohort study conducted at the Bascom Palmer Eye Institute, evaluating all glaucoma patients who underwent implantation of a Tube Extender between January 2015 and January 2023. Patients of any age were eligible for inclusion if they had documentation of Tube Extender use, identified either through surgical notes containing the terms “Tube Extender,” “tube extension,” or “New World Medical” or through implant device data indicating placement of a Tube Extender. For each patient, medical records were reviewed to collect demographic and clinical data, including age, sex, race, ethnicity, age at the time of tube extender implantation, type of procedure, original glaucoma drainage device, laterality, glaucoma diagnosis, days from the original glaucoma surgery to tube extender implantation, reason for surgery, procedure performed, and lens status prior to surgery. In addition, the first postoperative clinic notes were reviewed to confirm early postoperative status.

All analyses were performed in R (version 4.5.0) using RStudio. Continuous variables were summarized using means with standard deviations and medians with interquartile ranges, while categorical variables were reported as frequencies and percentages. Tables were generated directly in R using the *gtsummary* and *gt* packages. To explore whether the indication for Tube Extender use varied according to the type of glaucoma, a contingency table was constructed comparing glaucoma diagnosis and surgical indication, Fisher’s exact test was applied given the small sample size and sparse categories, and statistical significance was defined as *p* < 0.05.

### Surgical technique

**Fig. 1:**
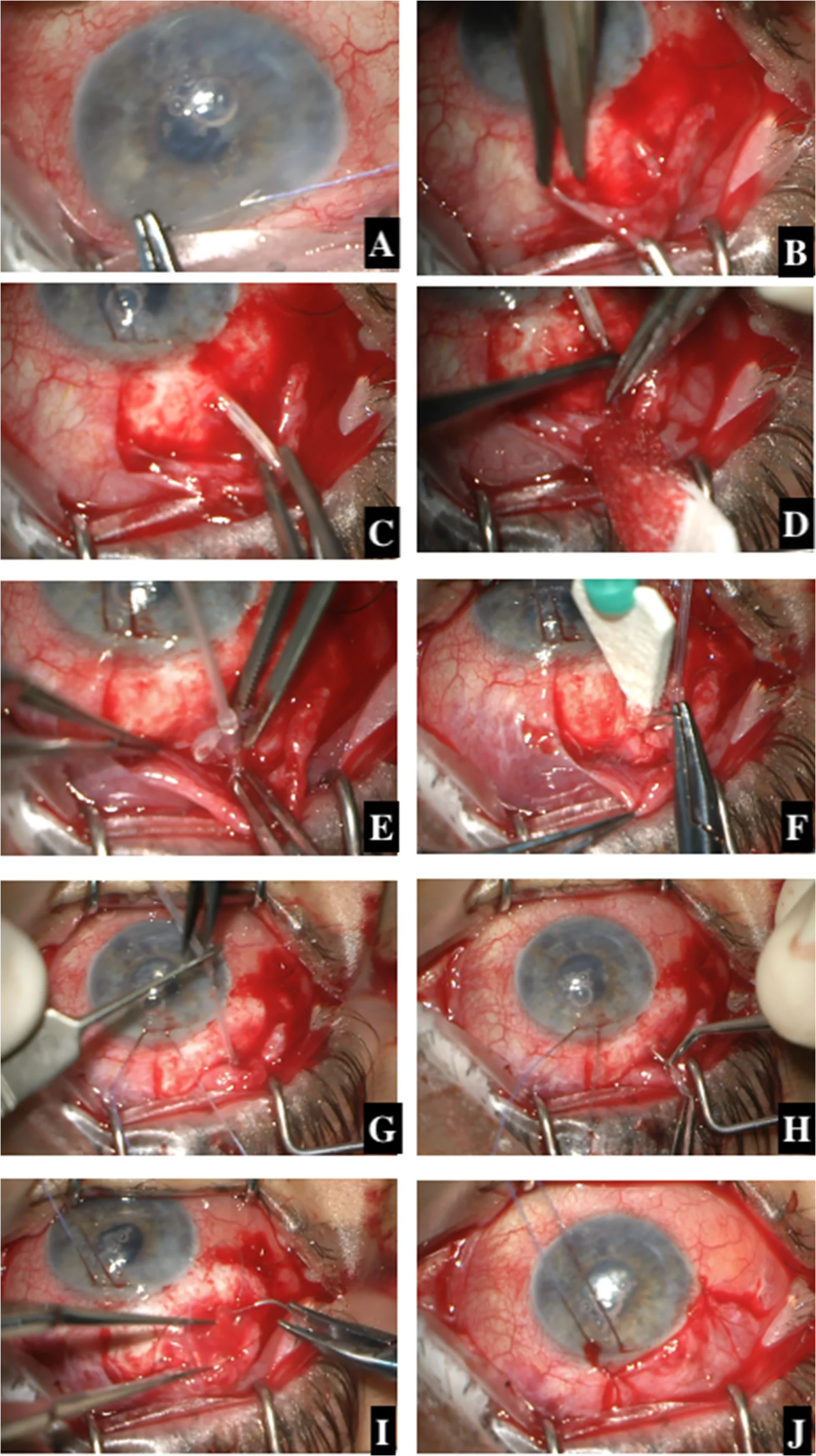
Surgical steps for implantation of a Tube Extender. (**A**) Corneal traction suture. (**B**) Conjunctival peritomy exposing the glaucoma drainage device plate and tube. (**C**) Removal of the tube from the eye. (**D**) Trimming of the original tube. (**E**) Attachment of the Tube Extender connector to the remaining segment. (**F**) Anchoring of the extender plate to the sclera with 10 − 0 polypropylene sutures. (**G**) Trimming of the new tube to the appropriate length. (**H**) Insertion of the beveled tip of the Tube Extender into the sulcus. (**I**) Placement of a patch graft over the exposed tube and Tube Extender plate. (**J**) Repositioning and suturing of the conjunctiva

A corneal traction suture is placed (Fig. 1A). Conjunctival peritomy to expose the glaucoma tube (Fig. 1B). Remove of original tube from anterior chamber or sulcus (Fig. 1C). Trimming the existing tube so that a short remnant (typically 2–4 mm) remains protruding from the plate (Fig. 1D). Slide the junction of the tube extender over the trimmed end of the original tube (Fig. 1E). Anchor the extender plate as posterior as possible by suturing its eyelets to the sclera using 10 − 0 polypropylene sutures (Fig. 1F) and trim the new tube extension to the appropriate length (Fig. 1G), typically with a beveled tip to facilitate insertion and minimize the risk of contact with the iris or cornea. Create a new scleral tract with a 23 -gauge needle or reuse the existing one into the desired location (such as the anterior chamber or sulcus), and insert the beveled tip of the extender’s tube through this tract (Fig. 1H). Place a patch graft over the exposed portions of the tube (both original and Tube Extender segments) to protect against conjunctival erosion (Fig. 1I), and reposition and suture the conjunctiva over the tube and graft to close the wound (Fig. 1J).

## Results

A total of 46 medical records referencing Tube Extenders were identified in surgical notes. Of these, only 22 operative notes documented actual implantation of a Tube Extender, and these cases were included in the final analysis.

Patient demographics and baseline characteristics were summarized in Table 1. The mean age at tube extender implantation was 53 (± 21 years), with a median of 58 years (IQR 40–66; range 6–82). The cohort was predominantly male (*n* = 15, 68.2%), with females comprising one third of the group (*n* = 7, 31.8%). The most common glaucoma diagnosis was primary open-angle glaucoma (*n* = 8, 36.4%), followed by uveitic glaucoma (*n* = 5, 22.8%) and neovascular glaucoma (*n* = 4, 18.2%). Less frequent diagnoses included chronic angle-closure glaucoma (*n* = 2, 9.1%), congenital glaucoma (*n* = 1, 4.5%), pseudoexfoliation glaucoma (*n* = 1, 4.5%), and secondary glaucoma due to prior surgery (*n* = 1, 4.5%). The single case of secondary glaucoma in this category was attributed to prior keratoplasty. Prior to tube extender implantation, half of the eyes were pseudophakic (*n* = 12, 54.5%), a third phakic (*n* = 8, 36.4%), and less than one tenth aphakic (*n* = 2, 9.1%). The majority of tube extenders were placed in eyes with Ahmed glaucoma drainage devices (*n* = 13, 59.1%), followed by Baerveldt devices (*n* = 6, 3%), one of which had a previous tube extension with a microcatheter, and a single case with a Molteno device (*n* = 1, 4.5%). In two cases (*n* = 2, 9.1%) the device type was unspecified. The mean interval between original glaucoma surgery and tube extender implantation was 764 days (± 1,330), with a median of 497 days (IQR 98–582).

**Table 1:** Patient characteristics. Baseline demographic and clinical features of eyes undergoing tube revision with tube extender implantation

Patient characteristics	
Age at tube extender implantation (years), Mean ± SD; Median (Q1, Q3)	53 ± 21; 58 (40, 66)
Sex, *n* (%)	
Male	15 (68%)
Female	7 (32%)
Type of glaucoma, *n* (%)	
POAG	8 (36%)
Uveitic glaucoma	5 (23%)
Neovascular glaucoma	4 (18%)
CACG	2 (9.1%)
Congenital glaucoma	1 (4.5%)
Pseudoexfoliation glaucoma	1 (4.5%)
Secondary to surgery	1 (4.5%)
Laterality, *n* (%)	
Right	14 (64%)
Left	8 (36%)
Eye lens status before surgery, *n* (%)	
Pseudophakic	12 (55%)
Phakic	8 (36%)
Aphakic	2 (9.1%)
Original type of glaucoma device, *n* (%)	
Ahmed	13 (59%)
Baerveldt	5 (23%)
Unspecified	2 (9.1%)
Baerveldt + angiocatheter tube extension	1 (4.5%)
Molteno	1 (4.5%)

The majority of tube revisions (*n* = 18, 81.8%) were performed for a single indication, while four cases (*n* = 4, 18.2%) were performed for two or more indications. The most frequent reasons for implantation were tube retraction (*n* = 9, 40.1%), corneal touch (*n* = 6, 27.3%), tube exposure (*n* = 5, 22.7%), and tube obstruction (*n* = 3, 13.6%) (Table 2). Of the three tube obstructions, two were caused by peripheral anterior synechiae and one by the iris itself. Notably, all cases of tube obstruction occurred in Baerveldt devices with pseudophakic eyes, while corneal complications, retraction, and exposure occurred in both Ahmed and Baerveldt implants, with no device-specific predominance. The single Molteno case was associated with tube exposure and retraction.

**Table 2:** Indications for tube revision. Primary reasons for tube extender implantation, listed by frequency

Reason for surgery, *n* (%)	
Tube retraction	6 (27%)
Corneal touch	5 (23%)
Tube exposure	4 (18%)
Need for corneal transplant	2 (9.1%)
Corneal touch and shallow anterior chamber	1 (4.5%)
Tube exposure and retraction	1 (4.5%)
Tube obstruction	1 (4.5%)
Tube obstruction and retraction from the sulcus	1 (4.5%)
Tube obstruction, corneal protection and retraction from the anterior chamber	1 (4.5%)

In our cohort, repositioning of the tube back into the anterior chamber was performed in more than half of the eyes (*n* = 14, 63.6%), followed by repositioning from the anterior chamber to the sulcus (*n* = 6, 27.3%), and repositioning within the sulcus (*n* = 2, 9.1%) (Table 3). Among the seven cases that required tube revision for cornea-related issues, six were managed by repositioning the tube from the anterior chamber to the sulcus, while in one case of corneal touch the tube was repositioned within the anterior chamber. Of the eight phakic eyes, seven had an Ahmed implant and one had a Baerveldt device that had already been fitted with a previous angiocatheter tube extension. Of the eight eyes in which the tube was ultimately positioned in the sulcus, seven were pseudophakic; the single phakic eye in this group was diagnosed with uveitic glaucoma and required a corneal transplant.

**Table 3:** Location of tube repositioning and associated procedures. Final tube position after tube revision and additional procedures performed in the same surgery

Location of tube repositioning and associated procedures	
Location of tube repositioning, *n* (%)	
Back into the anterior chamber	14 (64%)
From the anterior chamber to the sulcus	6 (27%)
Repositioning back into the sulcus	2 (9.1%)
Additional procedure(s), *n* (%)	
No	19 (86%)
Corneal transplant	2 (9.1%)
Cataract surgery	1 (4.5%)

We also assessed whether the indications for tube revision differed across glaucoma types. Indications such as tube retraction, obstruction, exposure, and corneal complications were observed across multiple diagnoses without a clear predominance. To formally test for an association, we constructed a contingency table of glaucoma type versus indication for tube revision and applied Fisher’s exact test. The result was not statistically significant (*p* = 0.21), suggesting that the underlying glaucoma diagnosis was unrelated to the indication for tube revision.

Review of the first postoperative clinic notes confirmed a good early postoperative status in all included cases.

## Discussion

In this retrospective cohort, Tube Extenders were used to address tube retraction, corneal-related issues, tube exposure, and tube obstruction, demonstrating the versatility of the device in managing tube position–related complications. These issues were effectively resolved by lengthening tubes that were either preoperatively too short or had to be trimmed during surgery, as confirmed in the early postoperative clinic notes.

Tube retraction can occur due to progressive tissue remodeling, eye growth in younger patients, or surgical miscalculation of initial tube length. Progressive tissue remodeling refers to contraction or fibrosis around the plate and anchoring tissue, leading to pulling back of the plate from its intended position [15]. In younger patients, eye growth may lead to retraction of the tube out of the anterior chamber and associated strabismus due to the smaller orbital volume [16]. The two pediatric patients in our cohort likely required tube revision related to eye growth, one due to tube retraction and the other due to corneal touch, and neither demonstrated strabismus.

For corneal issues, the Tube Extender facilitated the redirection of the tube away from the cornea. As seen in this and other studies, this can be done in cases of corneal touch, which may be detected by focal opacities, corneal edema, or signs of decompensation. Corneal touch is associated with progressive endothelial cell density loss, leading to corneal decompensation or keratopathy due to mechanical trauma to the endothelium. Although endothelial cell density loss after anterior glaucoma device insertion has been reported in several studies [17–20], including a comparative series including 62 eyes by Kim et al. showing a 25.6% loss with anterior chamber placement versus only 7.1% with sulcus placement of Ahmed glaucoma valve over two years [17], sulcus placement is rarely performed in phakic patients because of the risk of lens touch and cataract formation [21].

Tube Extenders were also used, as in our cohort, to manage tube exposures [12, 13, 22, 23], which can have serious consequences, including endophthalmitis [24–26]. A patch (cornea, sclera, pericardium, etc.) is essential to provide immediate structural coverage in cases of exposed tube [13, 25, 27]. Supporting this, a study by Alawi et al. of 43 eyes with primary tube exposure, four eyes were repaired using conjunctival closure without any patch graft; three of these subsequently re-exposed, leading the authors to conclude that repairs without patch grafting carry approximately double the risk of recurrence [22]. A systematic review by Neti et al. that included 118 cases of tube exposure repair reported that 86% of patients had no prior exposure repair, 9.6% had undergone one, 3.5% had two, and 0.9% had three previous repairs. The authors found that simple patch grafting was associated with a 13.1% re-exposure rate and an 88.4% 1-year survival, whereas rerouting the tube or placing it beneath a scleral flap or tunnel, always in combination with a patch graft, was associated with the highest survival, with no re-exposures observed during mean follow-up periods of 14 to 27 months.

In cases of tube obstruction, tube flushing or repositioning is sometimes required to redirect the tube bevel and restore aqueous flow. In doing so, it may be retracted from its desired position, necessitating lengthening. In our cohort the three obstructions were related with peripheral anterior synechiae and iris incarceration into the lumen, but obstructions can result from a variety of mechanisms. Membranous overgrowth, such as that seen in iridocorneal endothelial (ICE) syndrome, may proliferate over the tube tip and cause blockage [28]. Intraocular lens subluxation can also cause obstruction of the tube by the iris [29]. Tube kink [30], or proximal tube opening fibrosis may also cause tube obstruction [31], among others causes such as blood or vitreous [32, 33].

This study provides additional insights. Most revisions resulted in tubes being positioned from anterior chamber original placement back into the anterior chamber. In 58.3% of pseudophakic eyes, suggesting that the anterior chamber remains the preferred site when its anatomy permits in. Tube Extenders offered a versatile option for managing retraction, exposure, obstruction, and corneal complications across different glaucoma drainage devices. Their use preserved existing implants, minimized surgical trauma, and enabled individualized repositioning strategies. By presenting both patient characteristics and surgical details, this study adds context to the practical application of Tube Extenders in contemporary glaucoma surgery.

Limitations of the study include its retrospective design, which may introduce bias since data collection relies on existing medical records that may be incomplete or inconsistently documented, and potential confounding variables that may not have been captured. The modest sample size also reduces statistical power to detect meaningful associations between clinical factors. In addition, our study focused on reasons for tube revision with a Tube Extender, without evaluating long-term IOP control, device survival, or long-term complication rates of these interventions. Larger, multicenter studies with extended follow-up are needed to clarify the durability and comparative effectiveness of Tube Extender use relative to other available options for managing tube-related complications.

## Data Availability

The datasets generated and analyzed during the current study are available from the corresponding author upon reasonable request.
